# A Study on Doped Heterojunctions in TiO_2_ Nanotubes: An Efficient Photocatalyst for Solar Water Splitting

**DOI:** 10.1038/s41598-017-14463-0

**Published:** 2017-10-30

**Authors:** L. K. Preethi, Rajini P. Antony, Tom Mathews, Lukasz Walczak, Chinnakonda S. Gopinath

**Affiliations:** 1 Surface and Nanoscience Division, Materials Science Group, Indira Gandhi Centre for Atomic Research, Homi Bhabha National Institute, Kalpakkam, 603 102 India; 2 Chemistry Division, Chemistry Group, Bhabha Atomic Research Centre, Mumbai, 400 085 India; 3 PREVAC sp. z o.o., Raciborska 61, 44-362 Rogow, Poland; 4 Catalysis Division, National Chemical Laboratory, Dr. Homi Bhabha Road, Pune, 411 008 India

## Abstract

The two important factors that affect sunlight assisted water splitting ability of TiO_2_ are its charge recombination and large band gap. We report the first demonstration of nitrogen doped triphase (anatase-rutile-brookite) TiO_2_ nanotubes as sun light active photocatalyst for water splitting with high quantum efficiency. Nitrogen doped triphase TiO_2_ nanotubes, corresponding to different nitrogen concentrations, are synthesized electrochemically. Increase in nitrogen concentration in triphase TiO_2_ nanotubes is found to induce brookite to anatase phase transformation. The variation in density of intra-band states (Ti^3+^ and N 2p states) with increase in nitrogen doping are found to be critical in tuning the photocatalytic activity of TiO_2_ nanotubes. The presence of bulk heterojunctions in single nanotube of different nitrogen doped TiO_2_ samples is confirmed from HRTEM analysis. The most active nitrogen doped triphase TiO_2_ nanotubes are found to be 12 times efficient compared to pristine triphase TiO_2_, for solar hydrogen generation. The band alignment and charge transfer pathways in nitrogen doped TiO_2_ with triphase heterojunctions are delineated. Bulk heterojunctions among the three phases present in the nanotubes with intra-band defect states is shown to enhance the photocatalytic activity tremendously. Our study also confirms the theory that three phase system is efficient in photocatalysis compared to two phase system.

## Introduction

After the remarkable discovery of Asahi *et al*.^1^ on band gap engineering of TiO_2_ by nitrogen doping to absorb visible light, the effects of nitrogen^2–8^ and other non metals^9–13^ doping in various TiO_2_ nanomaterials are reported. It is found that compared to other non metals, nitrogen doping is more appropriate for extending the photocatalytic activity of TiO_2_ into visible region because of its comparable atomic size to that of oxygen, small ionization energy and stability^14,15^. These photo-absorption tuned TiO_2_ nanomaterials are utilized in many solar applications such as DSSC^16^, water splitting^2,17^, degradation of environmental pollutants^18^ etc.

The main drawback of nitrogen doped titania is that they suffer heavy electron-hole pair recombination as the additional extrinsic electronic levels introduced by doping can serve as recombination centres^19,20^. Several approaches are followed to overcome this drawback. One such approach is loading of noble metals with high electron affinity on TiO_2_ which effectively trap the photoinduced electrons for subsequent reduction reaction whereas the holes participate in the oxidation reactions. However, the noble metal loading is an expensive technique and also it has the disadvantage of possible separation of metal particles off the TiO_2_ support^21–24^. The other approaches include sensitizing TiO_2_ with organic dyes^25^, polymers^26^ and other narrow band gap inorganic semiconductors^27,28^. However these techniques face challenges such as, poor adhesion of dyes on TiO_2_ in aqueous media and degradation in corrosive as well as highly oxidizing environment^29^. Similarly the heterostructures formed by suitable narrow band gap semiconductors often undergo UV induced photocorrosion thereby limiting their application^29,30^.

It is known that titania exists as three polymorph viz. anatase, rutile and brookite which has different band structures^31^. The study on biphase heterostructured titania is reported to show the best photocatalytic efficiency due to the preferable alignment of band edges of the different phases^32^. Among biphase heterostructures, anatase-rutile biphase TiO_2_ has been widely studied and is proven to be more efficient compared to single phase TiO_2_^21,33^. There are also studies on other biphase structures such as anatase-brookite^34,35^ and rutile-brookite^36^. It is widely agreed that the biphase structure is effective compared to single phase in charge separation leading to higher efficiency in photocatalysis. Despite the fact that the heterostructures formed by two polymorphs of titania can endure photocorrosion, the composites are active only under UV light. Hence the strategy of coupling binary structure with nitrogen doping has been designed for high quantum efficient visible light photocatalysis, which is well received^21,37^.

The latest reports suggest that triphase TiO_2_ performs far better in charge pair separation compared to biphase heterojunction enhancing the photocatalytic efficiency^38–41^. The main drawback of the triphase system is that it is difficult to synthesize. Very few reports are available on the synthesis of triphase TiO_2_ and its applications. Although, there are many reports on nitrogen doped single and biphase TiO_2_, still there is lot of scope to be fulfilled to obtain a practicable photocatalyst. One such step towards this is to carry out nitrogen doping in triphase TiO_2_, to obtain an efficient visible light active photocatalyst. To the best of our knowledge there are no reports on study of nitrogen doping in triphase TiO_2_ for water splitting applications.

Recently our group reported a facile technique to synthesize triphase TiO_2_ where in all the three phases exist together in a single nanotube forming heterojunctions. It is found that the triphase TiO_2_ nanotubes have better efficiency compared to biphase and single phase nanotubes^41^. Our efforts to synthesize N-doped triphase TiO_2_ by annealing the triphase TiO_2_ in NH_3_ atmosphere resulted in N-doped biphase TiO_2_^21^. Efforts to synthesize N-doped triphase TiO_2_ at low temperatures through soft chemistry and electrochemical routes using various amines as nitrogen precursor resulted in destruction of tubular structure and carbon contamination. After several trials, use of carbon less precursor in combination with an electrochemical technique is identified. The phase transformation and defect formation with respect to nitrogen concentration is studied in detail. The photocatalytic activity of the nitrogen doped triphase TiO_2_ nanotubes in comparison to pristine triphase TiO_2_ nanotubes are evaluated by hydrogen generation through water splitting.

## Results

### Rapid Breakdown Anodization (RBA)

The synthesis of pristine TiO_2_ nanotubes are carried out by following Rapid Breakdown Anodization (RBA) technique as described in our previous report^41^ where the synthesis voltage is kept at 11 V (±0.03 V) to obtain anatase-rutile-brookite polymorph. Nitrogen doped triphasic TiO_2_ nanotubes (N-TiO_2_), corresponding to different nitrogen concentrations are synthesized by having 0.5, 1.5, 3 and 4.5 wt% hydrazine hydrate as nitrogen source in the 0.1 M HClO_4_ electrolyte solutions with 11 V (±0.03 V) as synthesis voltage. The pristine and doped TiO_2_ powders fallen into the electrolyte are collected, centrifuged, washed in double distilled water and dried in air atmosphere overnight. The dried samples are labeled as, T-ARB for anatase-rutile-brookite TiO_2_ nanotubes, whereas labels NT-0.5, NT-1.5, NT-3 and NT-4.5 represent the N-doped TiO_2_ nanotubes synthesized with 0.5, 1.5, 3 and 4.5 wt% of nitrogen source respectively. The nitrogen concentration present in NT-0.5, NT-1.5, NT-3 and NT-4.5 are estimated to be 0.19, 0.29, 0.37 and 0.48 At % respectively from N 1s spectra of XPS which is demonstrated in later sections.

### Phase transformation analysis

**Figure 1:**
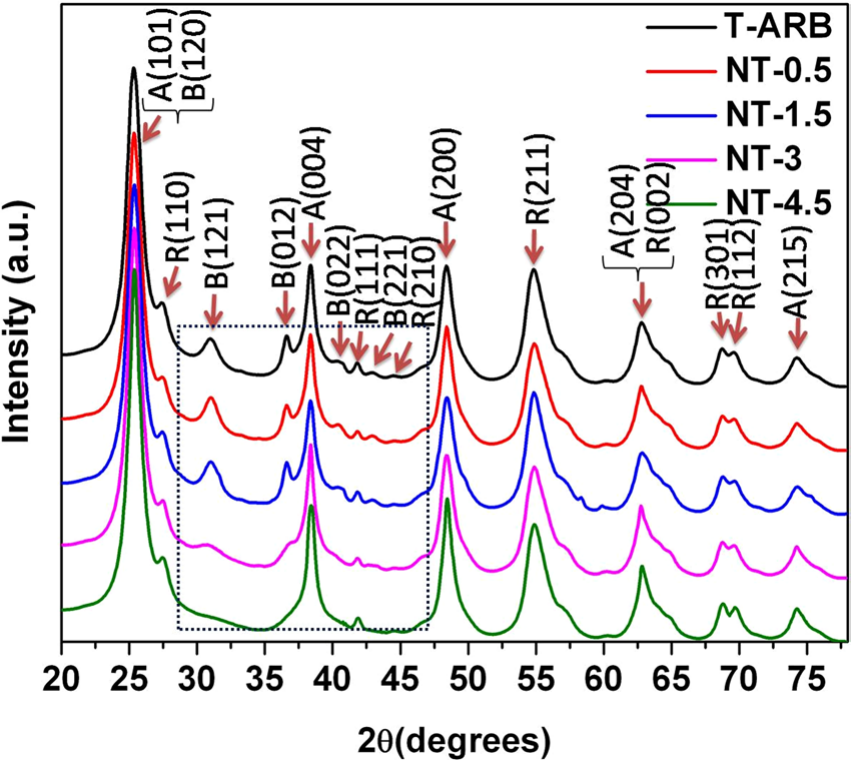
X-Ray diffraction patterns of pristine and N-TiO_2_ nanotubes.

The XRD patterns of the pristine and N-doped catalysts are shown in Fig. 1. The characteristic XRD peaks of the samples are matched with the corresponding diffraction features of anatase, rutile and brookite phases (JCPDS files: Anatase: 21–1272; Rutile: 21–1276; and Brookite: 29–1360). It is observed that all the three phases are present in pristine T-ARB. The peak intensities corresponding to different phases obtained for samples synthesized at different concentrations of nitrogen precursor are found to be different. It is noticed that, as we increase the nitrogen source concentration from 0.5 wt to 4.5%, the intensity of brookite peaks remain unaltered in NT-0.5 and NT-1.5 whereas it gradually decreases in NT-3 and finally disappears in NT-4.5. On the other hand, the anatase peaks grows in intensity with increase in nitrogen concentration. The brookite to anatase phase transformation with increase in nitrogen doping, resulting in ARB to AR transformation in N-TiO_2_ nanotubes is evident from Fig. 1 (the window depicted). The individual phase composition of each sample is determined using the following equations^42^:

1 $$Wa=KaAa/(KaAa+Ar+KbAb)$$

2 $$Wr=Ar/(KaAa+Ar+KbAb)$$

3 $$Wb=KaAa/(KaAa+Ar+KbAb)$$

where *Wa, Wr* and *Wb* represent mass fractions of anatase, rutile and brookite, respectively; *Aa, Ar* and *Ab* represent the integrated intensities of anatase (101), rutile (110) and brookite (121) phases respectively. *Ka* and *Kb* stands for correction coefficients which have values of 0.886 and 2.721 respectively^42^. A numerical technique is used to deconvolute the overlapping anatase (101) and brookite (120) peaks. Phase quantification and grain size obtained from Scherrer equation, using the characteristic XRD peaks of anatase A(101), rutile R(110) and brookite B(121) are tabulated in Table 1. It is found that T-ARB is composed of 45% anatase, 26% rutile and 29% brookite phases. Marginal increase in grain size is noticed in case of rutile phase (almost in the same range), whereas reasonable increase of grain size in anatase phase with decrease in brookite grain size is noticed with increase in nitrogen concentration. The cause of brookite to anatase phase transformation can be attributed to the relation between phase stability and crystal size viz. anatase is more stable at crystallite sizes below 11 nm, brookite at crystal sizes between 11 and 35 nm, and rutile with the crystal sizes ≥ 35 nm^42^. The initial particle sizes of brookite and anatase in the pristine triphase TiO_2_ nanotubes are 6.7 and 5.1 nm, respectively. Upon nitrogen doping, particles of all the three phases coarsen. Due to this coarsening effect, the particle size of every phase increases initially. However, as the nitrogen doping concentration increases, the smaller crystallite size of brookite (5.3 nm) makes it unstable and is converted to anatase which is more stable phase at crystallite sizes between below 11 nm. The origin of the transitions can be attributed to the different surface energies of anatase and brookite^43^. Most nanocrystalline materials have metastable structure which gets converted to stable structure with change in temperature, pressure, grain growth, defects etc^44–47^. In case of TiO_2_, anatase is stable at low temperature whereas rutile is stable at high temperatures^44^. Brookite, on the other hand is metastable when compared to rutile and anatase^48^. Therefore it is expected that brookite should transform either to anatase or rutile. Since the surface Gibbs free energy of anatase is lower than that of rutile phase^49,50^ when the particle size is small, brookite prefers to stabilize by transforming into anatase phase rather than into rutile phase. Pen and Banfield *et al*.^51^ showed that the brookite phases can be considered as a polytype (reversible solid state transformation) of anatase, constructed out of essentially identical octahedral layers stacked in different ways. Therefore the inter conversion of anatase to brookite or brookite to anatase can be invoked by the displacement of Ti atoms into adjacent sites in the lattice. When the concentration of nitrogen doping into the lattice increases, the induced strain in the lattice causes displacement of lattice atoms. In addition, the oxygen vacancies generated by N-doping also leads to Ti displacement. These in turn invoke the unstable brookite to transform to stable anatase phase as it is the more stable phase in the size range of <11 nm.

**Table 1:** Quantification and crystallite size of individual phases in TiO_2_ and N-TiO_2_ polymorphs.

Samples	Concentration of Nitrogen (At %)	Anatase		Rutile		Brookite	
		Phase %	Size nm	Phase %	Size nm	Phase %	Size nm
T-ARB	—	45	6.7	26	6.23	29	5.19
NT-0.5	0.19%	46	6.9	25	6.22	29	5.26
NT-1.5	0.29%	54	7.2	26	6.191	20	5.03
NT-3	0.37%	65	7.6	27	6.465	8	4.42
NT-4.5	0.48%	72	8.06	28	6.68	—	—

**Figure 2:**
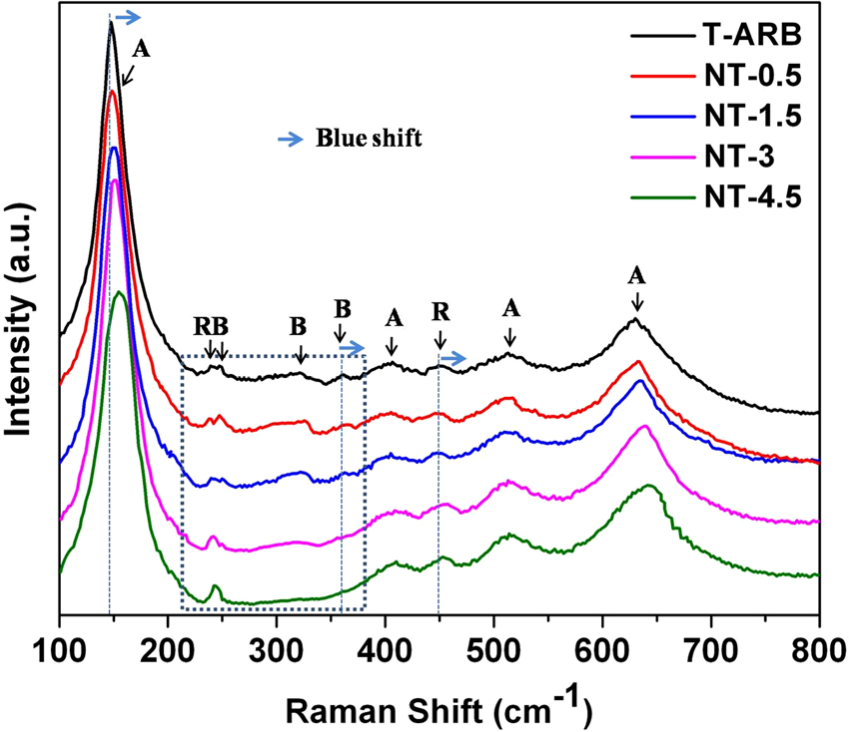
Raman Spectra of pristine and N-TiO_2_ nanotubes.

The phase compositions of pristine and N-TiO_2_ nanotubes are further examined by Raman spectra analysis (Fig. 2). The characteristic Raman modes of anatase phase appear at 148, 402, 515 and 630 cm^−1^ for T-ARB and with slight shift in N-TiO_2_ polymorphs. These peaks are assigned to E_g_, B_1g_, A_1g_/B_1g_ and E_g_ modes of anatase phase respectively^3^. The peak corresponding to E_g_ mode of rutile appears around 448 cm^−1^ ^52^ and a small peak around 240 cm^−1^, as a result of second order scattering effect in rutile phase^52^, also appears in all the samples without and with shift due to N doping. Similarly, the Raman features corresponding to brookite phase around 245, 322 and 360 cm^−1^ representing A_1g_, B_1g_ and B_2g_ modes respectively^53^ are clearly visible in T-ARB and with slight shift in NT-0.5 and NT-1.5; whereas their intensities decreases in NT-3 and are absent in NT-4.5. Thus Raman studies further confirms the ARB to AR phase transformation with increase in nitrogen doping which is clearly depicted in the window of Fig. 2. It is observed that the peaks corresponding to anatase, rutile and brookite modes are blue shifted (the shift is depicted in Fig. 2) as a result of nitrogen doping. The shift of the peaks can be attributed to change in size^54,55^, lattice disorder or defects such as oxygen vacancies^56,57^. The blue shift is also indicative of better electronic interaction between titania and nitrogen doping^24^.

**Figure 3:**
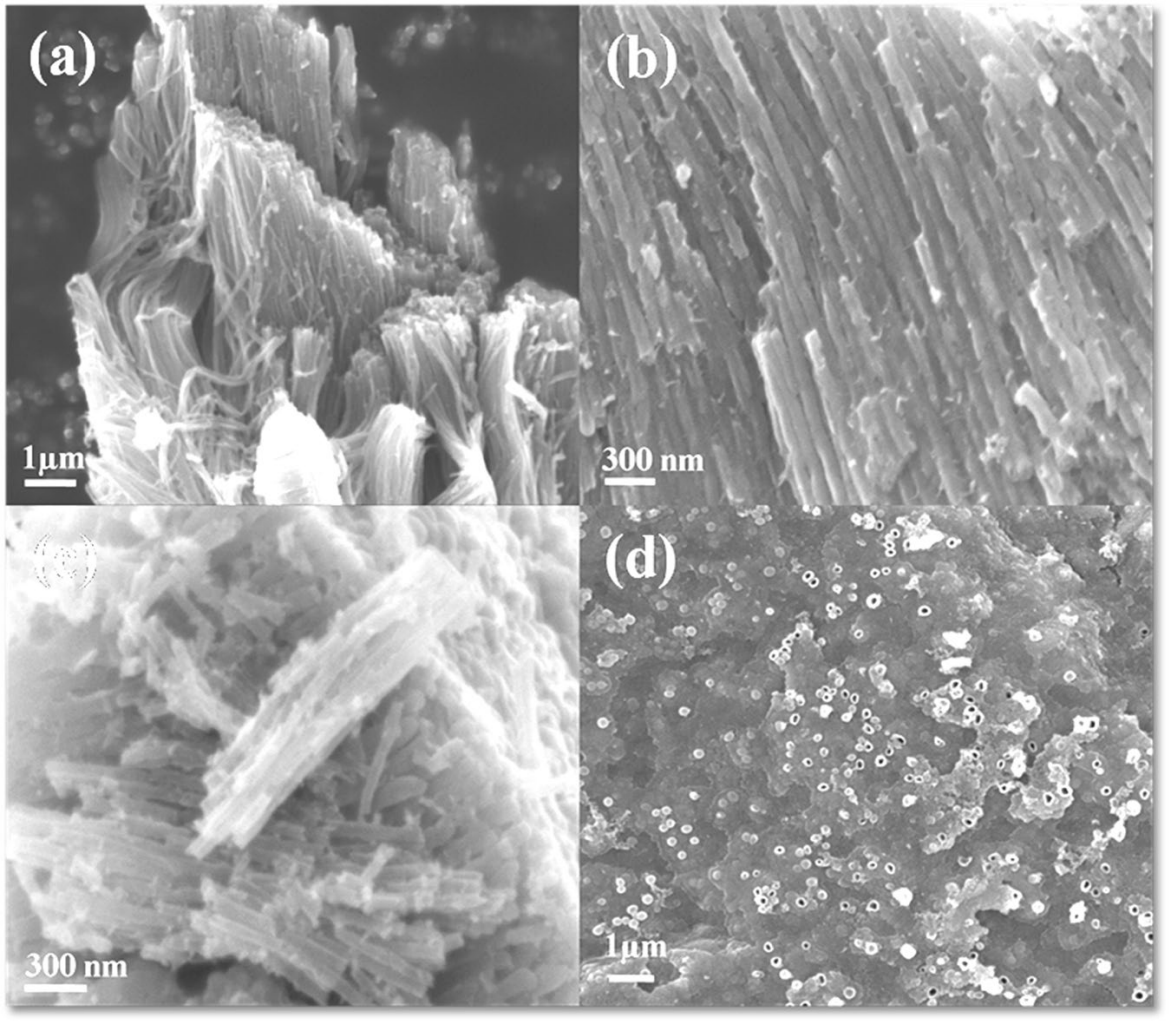
FESEM images of (**a**) T-ARB, (**b**) NT-1.5, (**c**) NT-4.5 and (**d**) perpendicular view of NT-1.5 nanotubes.

The surface morphology of pristine and nitrogen doped TiO_2_ is analyzed using FESEM and TEM on selected samples. The tubular morphology is clearly visible in the FESEM images (Fig. 3a–c) which represent the surface of T-ARB, NT-1.5 and NT-4.5. The tubular diameters are observed to be in the range of 30 to 120 nm for all the samples. Figure 3d shows the perpendicular view of the nanotubes present in NT-1.5 samples. Microchemical investigations were carried out for one of the nitrogen doped triphase TiO_2_ nanotubes samples by energy dispersive X-ray (EDX) analysis in order to show the elemental distribution. Figure S1a in the supporting information shows the secondary electron image of NT-1.5. The corresponding X-ray map for the characteristic emission of Ti K_α_, O K_α_ and N K_α_ are shown in Figure S1b–d respectively. The maps clearly show the uniform distribution of elements (Ti, O and N) in TiO_2_ nanotubes. Although quantification of low Z elements using the EDS analysis is not reliable, in line with our analysis, fewer signals from N K_α_ (Figure S1d) indicate the presence of low concentration of nitrogen in TiO_2_ nanotubes, suggesting that it is being doped.

**Figure 4:**
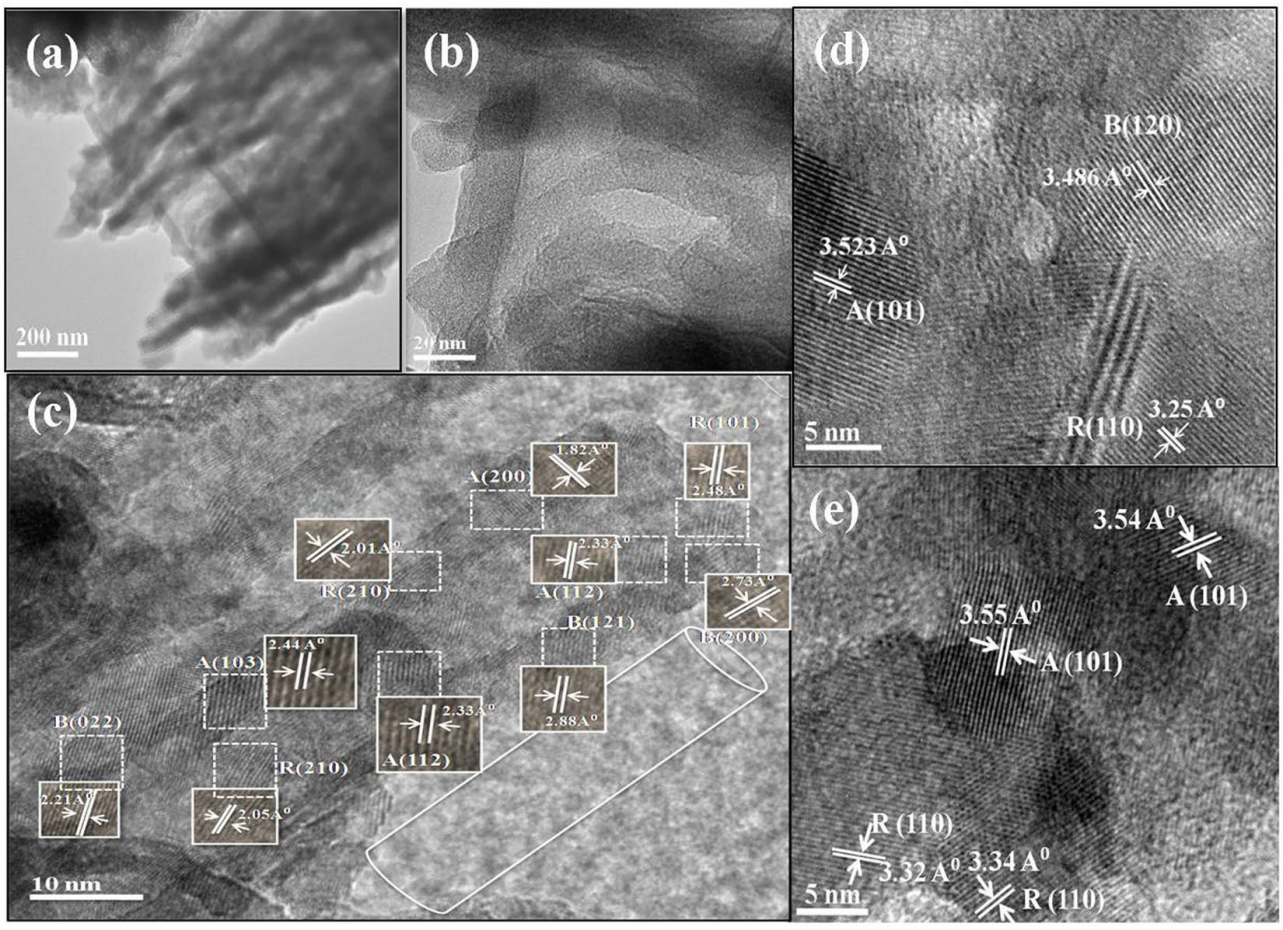
Bright field TEM image: (**a**) T-ARB and (**b**) NT-1.5; HRTEM images: (**c**) T-ARB (**d**) NT-1.5 and (**e**) NT-4.5.

The bright field image given in Fig. 4a and b also confirms the tubular morphology of T-ARB and NT-1.5. From HRTEM analysis of single nanotube of T-ARB (Fig. 4c), it is evident that the three phases viz anatase, rutile and brookite are present in a single nanotube. The triphase nature with well resolved lattice features is retained in NT-0.5, NT-1.5, NT-3 as evident from the HRTEM analysis represented in Fig. 4c and d. The interplanar spacing corresponding to brookite phases is not frequently observed in close proximity in HRTEM images of NT-3, whereas it is completely absent in the case of NT-4.5 (Fig. 4e). This further confirms that the TiO_2_ nanotubes undergoes phase transformation from ARB to AR composition with increase in nitrogen doping, which corroborate with results obtained from XRD and Raman spectra analysis. An important observation is the hetero-junctions formed between various phases of titania in the nanotubes. This demonstrates the physical connectivity between three phases at nanoscopic levels, and the junctions are expected to act as charge separation centers.

### Band gap analysis of TiO_2_ and N-TiO_2_ nanotubes

**Figure 5:**
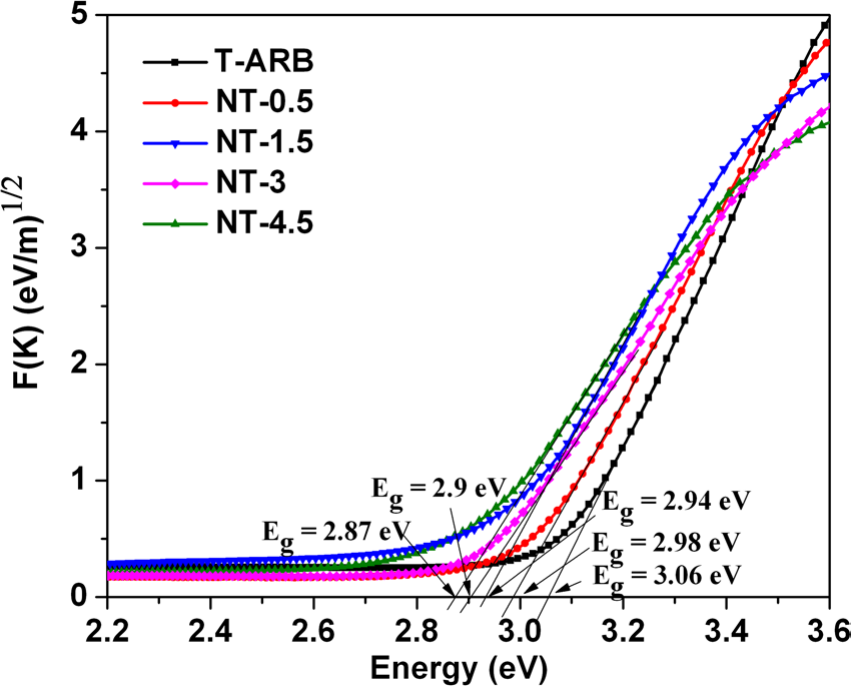
Kubelka-Munk plot of T-ARB and N-TiO_2_ nanotubes.

The optical properties of the synthesized pristine and N doped TiO_2_ nanotubes were analyzed using UV-visible Diffuse Reflectance Spectroscopy (DRS) (Fig. 5). The Kubelka-Munk (KM) function corresponding to the DRS spectra is denoted as

4 $$F(K)={(\alpha hv)}^{1/n}=A(hv-{E}_{g})$$

where *α*, *υ*, *A* and *E*_*g*_ are absorption coefficient, light frequency, proportionality constant and band gap respectively. The constant value of n is equal to ½ or 2, depends on the type of semiconductor. Assuming TiO_2_ as an indirect band gap semiconductor, the band gap can be evaluated from the plot of *F(K)* = *(αhυ)* ^*1/*2^ vs *hυ*, by extrapolating the straight line to the x-axis. Band gap of pristine TiO_2_ nanotubes (T-ARB) is found to be around 3.06 eV. The degree of nitrogen doping strongly affects the visible light absorption of TiO_2_ leading to monotonic decrease in band gap with increasing nitrogen doping. The lowest band gap of 2.87 eV is achieved in NT-4.5 which has highest nitrogen doping.

### Hydrogen generation by Photocatalytic Water Splitting

**Figure 6:**
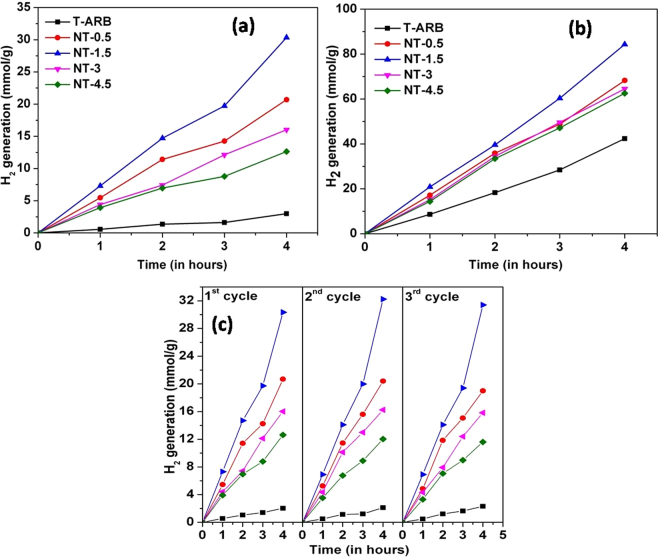
Hydrogen generation by (**a**) pristine T-ARB and N-TiO_2_ nanotubes; (**b**) Pt loaded T-ARB and N-TiO_2_ nanotubes, and (**c**) verification of stability of photocatalysts under one sun conditions.

The water splitting experiments are carried out for all the photocatalysts in water/ethanol mixture under one sun conditions. The plot in Fig. 6a shows that the volume of hydrogen generation of TiO_2_ polymorphs increased after nitrogen doping. The hydrogen generation efficiency of the given photocatalyst is in the order of NT-1.5 > NT-0.5 > NT-3 > NT-4.5 > T-ARB. The maximum hydrogen generation of 30.2 mmol/g is produced by NT-1.5 after 4 hours of light illumination. It is already noted that NT-0.5, NT-1.5 and NT-3 possess triphasic nature whereas NT-4.5 is biphasic. Sample NT-1.5 exhibits better efficiency compared to NT–0.5 because of higher nitrogen content. The lower efficiency of NT-3 compared to NT-0.5 and NT-1.5 despite its higher nitrogen doping is attributed to its low three phase content. The brookite content in NT-3 is very less that it acts almost like biphase TiO_2._ The water splitting efficiency is lowest for NT-4.5 which is attributed to the absence of brookite phase; which likely leading to lower charge separation/diffusion because of lower number of bulk heterojunctions and hence low activity.

In order to distinguish the water splitting efficiency with respect to co-catalyst loading, Pt nanoparticles are loaded on T-ARB and N-TiO_2_ samples using the procedure reported^21^. The highest hydrogen generation efficiency is obtained by Pt loaded NT-1.5 (70 mmol/g) which is nearly double to that generated by Pt loaded T-ARB (36 mmol/g) (Fig. 6b). This can be attributed to high visible light absorption in nitrogen doped sample.

Even though visible light absorption exists in other N-TiO_2_ polymorphs, the hydrogen generation by these samples after Pt loading is low proving that the nitrogen doping is in optimum level in NT-1.5. The hydrogen generation efficiency of Pt loaded NT-0.5, NT-3, and NT-4.5 are almost in same range. However we see the trend NT-0.5 ≥ NT-3 > NT-4.5 is maintained in Pt loaded samples. This can be attributed to the difference in phase composition (Table 1) (as NT-0.5 and NT-3 possess triphase composition whereas NT-4.5 possesses biphase composition).

To verify the reusability and the long term stability of the photocatalyst, H_2_ generation measurements were carried out in 3 cycles (Fig. 6c). All three cycles shows the same trend as well as the same range of values of hydrogen generation for different photocatalysts. Thus, reusability of the photocatalysts is discerned from the results.

### Charge transfer Analysis

**Figure 7:**
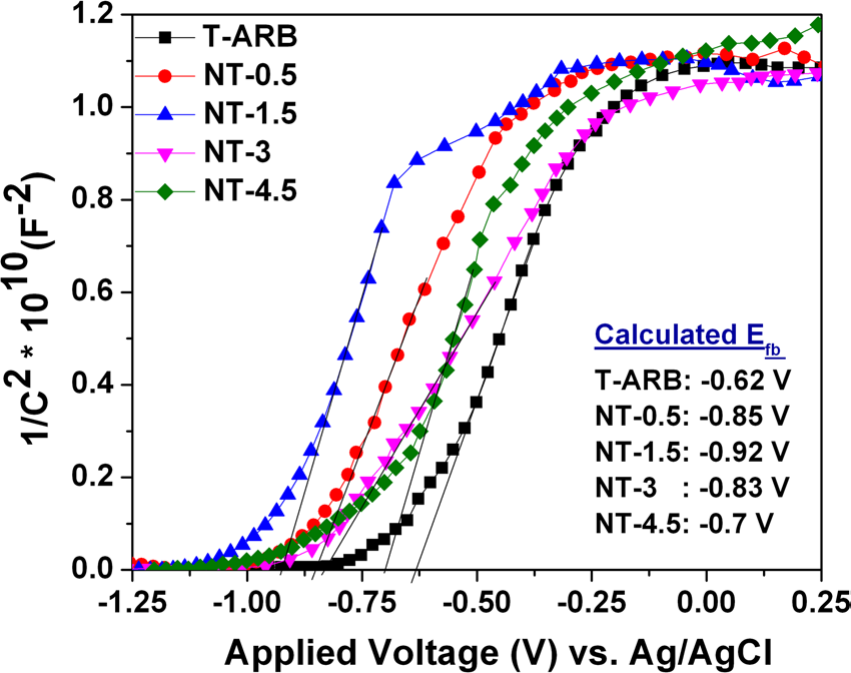
Mott Schottky plots of T-ARB and N-TiO_2_ nanotubes.

To ascertain the improvement in charge separation due to band alignment in multiphase system, Mott-Schottky analysis is performed under UV light for pristine and nitrogen doped TiO_2_ nanotubes in 0.5 M Na_2_SO_4_ electrolyte. Figure 7 represents the Mott-Schottky plots, from which the flat potential E_fb_ is calculated using the Mott-Schottky relation^21^

5 $${({C}_{cs})}^{-2}=\frac{2(E-{E}_{fb}-\frac{kT}{e})}{{N}_{D}\varepsilon {\varepsilon }_{o}e{A}^{2}}$$

where, ${{C}}_{{sc}}$ = space charge capacity, ${N}_{D}$ = charge carrier density of the sample, $\varepsilon$ = dielectric constant of the sample (100 for TiO_2_ nanotubes), *ε*_*o*_ = permittivity in vacuum and *A* = active surface area. ${E}_{{fb}}$ is calculated from the intercept extrapolating a straight line from *1/C* ^*2*^ versus potential plot. All samples exhibit a positive slope in Mott schottky plots, as expected for n-type semiconductors. The observed cathodic shift in flat band potential is in the order of NT-1.5 > NT-0.5 > NT-3 > NT-4.5 > T-ARB. The hydrogen generation by water splitting also follows the same trend (Fig. 6). When TiO_2_ is irradiated, the upward shift of the Fermi level enhances the charge separation at the semiconductor/electrolyte interface leading higher degree of band bending at the TiO_2_ surface compared to that in dark thus leading to efficient charge separation. The resultant electrons in conduction band and holes in the valence band take part in the redox reaction^58,59^.

In a multi-phase system, under UV illumination, the excited charge carriers transfer from one phase to another leading to a higher shift in the Fermi level towards the conduction band edge. The degree of Fermi level shift depends on the number of phase junctions present^41^. In present case, shift in the Fermi level is contributed by two factors viz. N doping and phase composition. The T-ARB sample exhibits low flat band potential among all the samples after irradiation, because the Fermi level shift is contributed only by triphase composition as there is no N-doping. As we can see from the flat band potential values, the nitrogen doped triphase samples such as NT-0.5, NT-1.5 and NT-3 possess same range of values compared to N-doped biphase NT-4.5. This indicates that the charge separation is better in case of nitrogen doped triphase samples.

The highest flat band potential is observed in NT-1.5 which is the best photo active sample as discerned from water splitting experiments. The crystallite size analysis indicates NT-1.5 possess overall smaller crystallite size of anatase, rutile and brookite compared to other doped triphase samples (NT-0.5 and NT-3). This smaller crystallite size would lead to more number of bulk heterojunctions and is expected to assist in effective charge separation. This reflects in the Mott-Schotky plots presented above (NT-1.5 > NT-0.5 > NT-3). Further, the presence of all three phases in close proximity in NT-1.5, as observed in the HRTEM analysis (Fig. 4c and d), makes it an efficient photocatalyst for water splitting due to the presence of bulk heterojunctions.

### XPS analysis

**Figure 8:**
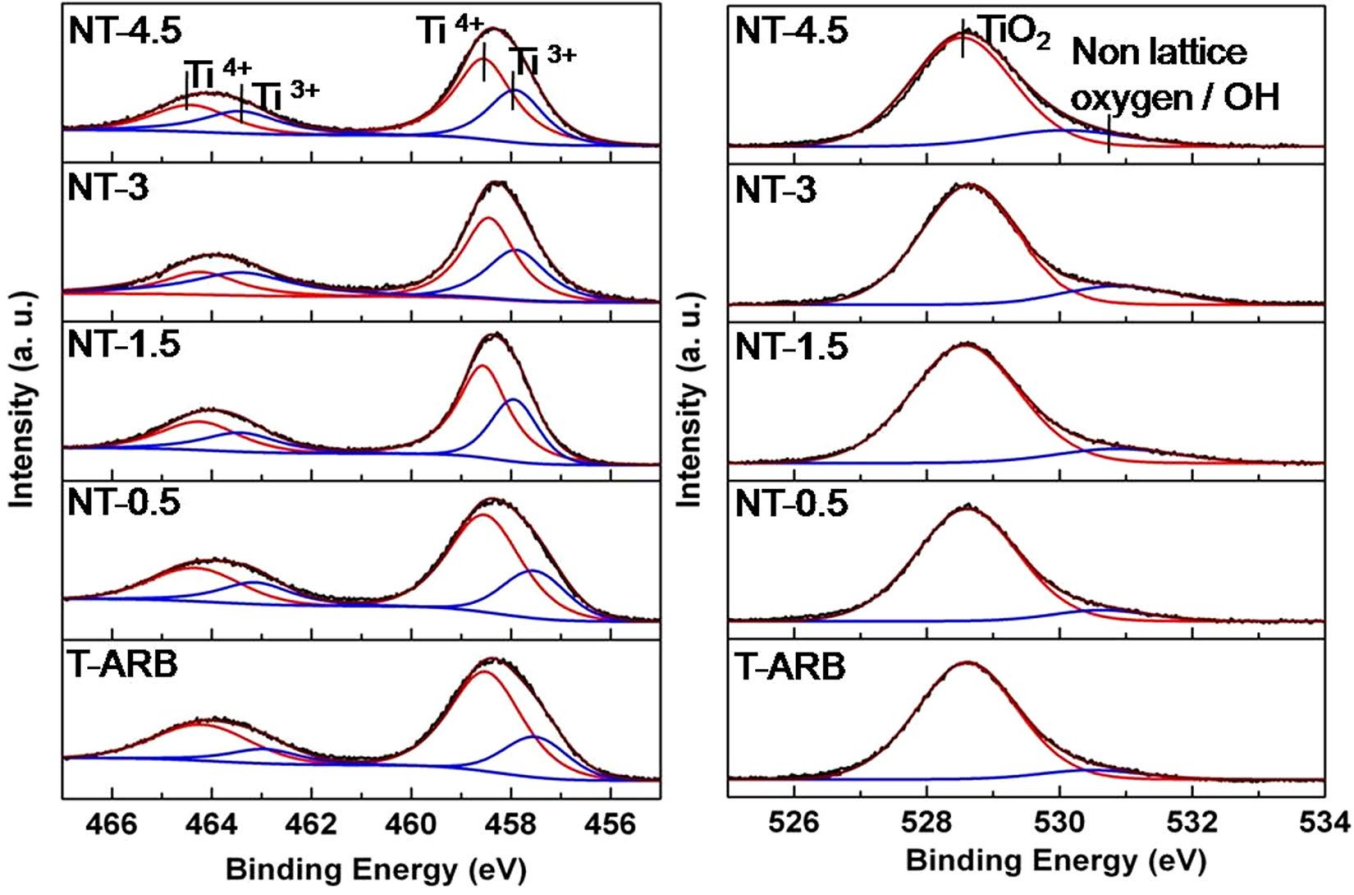
Deconvolution of (**a**) Ti 2p spectra and **(b**) O 1 s spectra of pristine and N doped TiO_2_ polymorphs.

To investigate the change of surface bonding of TiO_2_ nanotubes induced by nitrogen doping, XPS analysis is performed for all the samples which are deconvoluted and presented in Fig. 8. The Ti 2p core level spectrum of all samples, deconvoluted using CasaXPS, exhibited Ti 2p spin-orbit doublet centered at binding energies 458.6 and 464.1 eV, that corresponds to Ti 2p_3/2_ and Ti 2p_1/2_ core levels of Ti^4+^ species, respectively. Our observation is in agreement with the literature^60,61^. In addition, the presence of shoulder peaks around 456.8 and 462 eV indicate the presence of Ti^3+^ in pristine T-ARB as well as in N-TiO_2_ samples. The area of Ti^3+^ peak increases with increase in N doping by 7.8% in NT-0.5, 13% in NT-1.5, 23% in NT-3 and 24.7% in case of NT-4.5 compared to that in T-ARB. The localization of charge carriers generated due to oxygen vacancy formation at Ti^4+^ could be the cause for the reduction of Ti^4+^ to Ti^3+^.

**Figure 9:**
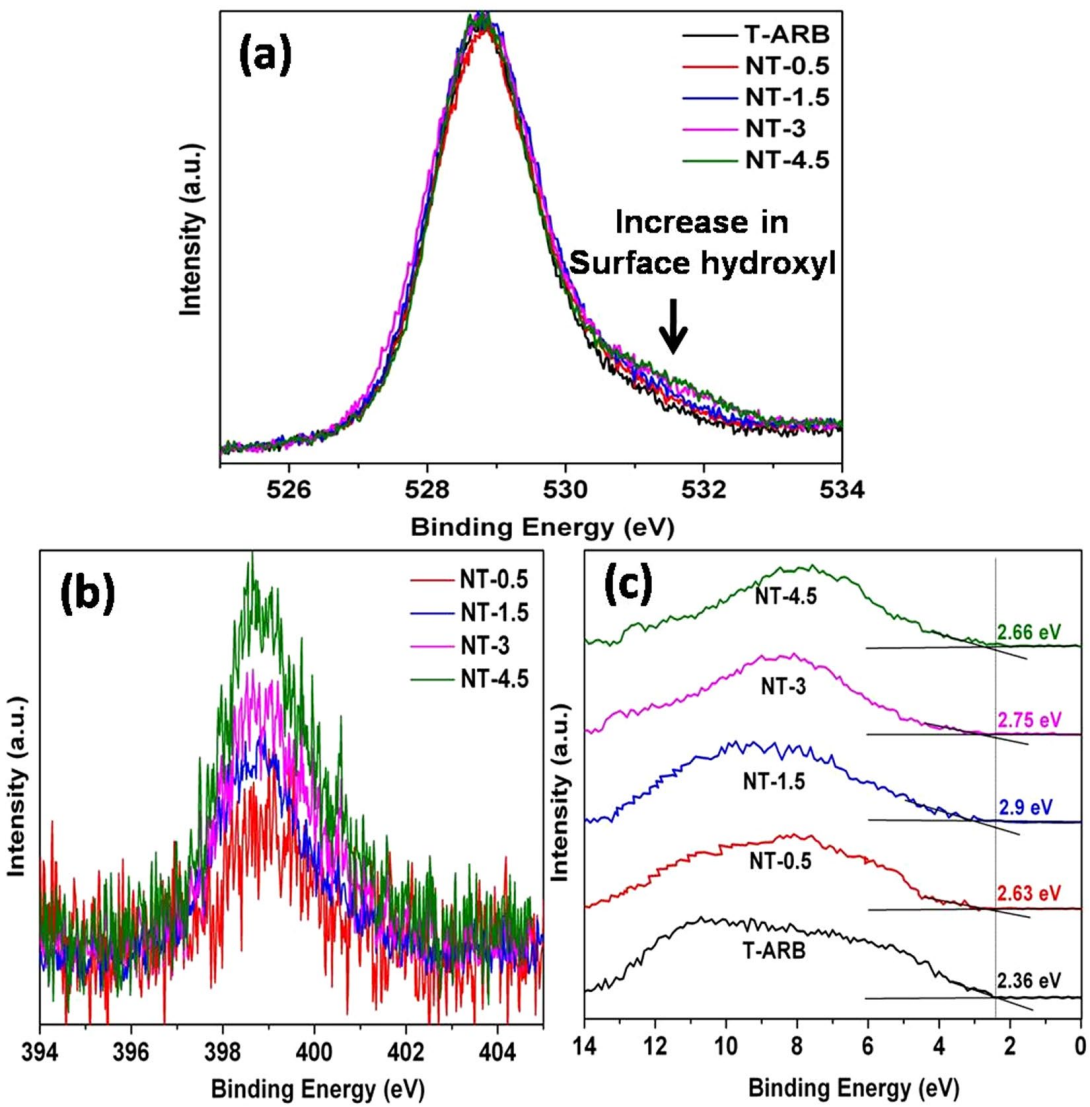
(**a**) O 1 s spectra, (**b**) N 1s spectra and (**c**) UPS analysis of pristine and N doped polymorphs.

The O1s spectra of all the samples are deconvoluted into two peaks which are centered at 529.8 and 531 eV which are represented in Fig. 8b. The O1s spectra of pristine TiO_2_ centered around 529.8 eV correspond to the lattice oxygen. In addition to this lattice oxygen signature peak, a shoulder can be discerned at higher binding energies, whose area increases with increase in nitrogen doping (Fig. 9a). The shoulder peak at 531 eV occurring in nitrogen doped polymorphs is attributed to the surface hydroxyl group (Ti-OH)^21^. The quantity of OH species increases initially with increase in nitrogen doping however it remains unaltered at higher degree of nitrogen doping as in the case of NT-3 and NT-4.5 (Fig. 9a). The area of the peak at 531 eV increases by 6% in NT-0.5, 11% in case of NT-1.5 and 19% (average) in case of NT-3 and NT-4.5 respectively compared to that in T-ARB.

Incorporation of nitrogen into the lattice results in the formation of oxygen vacancies in order to maintain charge neutrality. The oxygen vacancies at the surface take the form of missing oxide ions in the bridging oxygen row (bridging oxygen vacancies in the form: Ti^3+^ - V_o_ - Ti^3+^ where V_o_ denotes the oxygen vacancy) leading to the formation of co-ordinatively unsaturated Ti^3+^ ions. These Ti^3+^ ions have distorted coordination geometry, because of adjacent oxide ion vacancies. They have a tendency to form additional coordination and therefore, the oxygen vacancies act as adsorption sites for organic and water molecules thereby playing an important role in the photo-oxidation^62^.

When water molecules come in contact with nanotube surface, they are adsorbed at the bridging oxygen vacancies to form Ti – OH - Ti bridge^62,63^. The bridging hydroxyl groups are in close proximity to adsorbed water molecules and hence form, through hydrogen bonding, hydrated bridging OH^64^. The hydrated bridging OH groups offer a channel for the transfer of photo-generated holes, giving rise to 2 H^+^, Ti-OH and Ti-O^−^ species^64^. The generated H^+^ species are reduced to H_2_ by the photo-generated electron and the two adjacent Ti-O^−^ species couple to form surface peroxide. With increase in nitrogen doping, oxygen vacancy concentration increases which in turn increases hydrated bridging OH group resulting in enhanced hole assisted photo-catalytic reaction. However, in the present study, the expected trend is not observed. An initial increase in hydrogen generation with increase in N-doping followed by decrease in hydrogen generation is observed. The initial increase in hydrogen generation can be attributed to the increase in light absorption due to nitrogen doping, easy charge transfer across the bulk heterojunctions due to the inter band states, in addition to the increase in surface hydroxyl group. Although, the increase in nitrogen concentration increases the photo-absorption and surface hydroxyls, the subsequent reduction in hydrogen generation with increase in N-doping is observed. This can be attributed to the decrease in three phase content with increase in N-doping and only two phases (anatase-rutile) are present at highest N-concentration (NT-4.5). This shows that in N-doped samples, the effect of phase content overshadows the effect of surface hydroxyl group.

Presence of N-doping can be understood from the high resolution N 1s spectra of the N doped samples which are represented in Fig. 9b. The peaks are positioned around 398.7 in all the samples which correspond to doped nitrogen, which is consistent with our previous report and other reports found in literature^65–68^. The N 1s peak intensity gradually increases from NT-0.5 to NT-4.5 which confirms that the quantity of nitrogen doping in TiO_2_ crystal increases with increase in hydrazine hydrate concentration. The nitrogen content in NT-0.5, NT-1.5 and NT-4.5 are calculated to be of 0.19%, 0.29%, 0.37% and 0.48 At% respectively. In order to identify the effect of nitrogen doping on valence band offsets, UPS analysis was performed for all the samples. Figure 9c shows the UPS spectra of both pristine and N-TiO_2_ samples. The obtained VB edge values of the samples are listed in label of Fig. 9c. It is noticed that as we increase the nitrogen content, the gap between the valence band edge to Fermi level increases from T-ARB until NT-1.5, whereas it starts to decrease with the further increase in nitrogen doping (as for the case of NT-3 and NT-4.5). However it is noticed in band gap analysis from DRS that the band gap decreases with nitrogen doping. The results indicate that the Fermi level shifts towards conduction band edge until the doping is at 0.29% (NT-1.5), then it starts to shift away from the conduction band edge as in the case of NT-3 and NT-4.5 where the nitrogen doping is high. This can be attributed to the change in phase composition upon nitrogen doping which changes the Fermi level position^41^. As in the case of low nitrogen doping, the Ti^3+^ defects states and N 2p density start to increase. At higher nitrogen doping, it is observed that the Ti^3+^ defects are unaltered whereas the N 2p states grow denser. This should in turn decrease the gap between the Fermi level and VB edge at higher N doping. However it is not observed so in our case. This is due to the presence of multiple phases, where the Fermi level is near to conduction band edge in triphase system and it start to shift away from CB edge when the triphase system is converted to biphase system. The results coincide with the Mott-Schottky results where we see the similar pattern in flat band potential. Thus phase composition plays a major role in water splitting reactions as triphase increases the charge pair separation compared to biphase system.

### Photoluminescence

**Figure 10:**
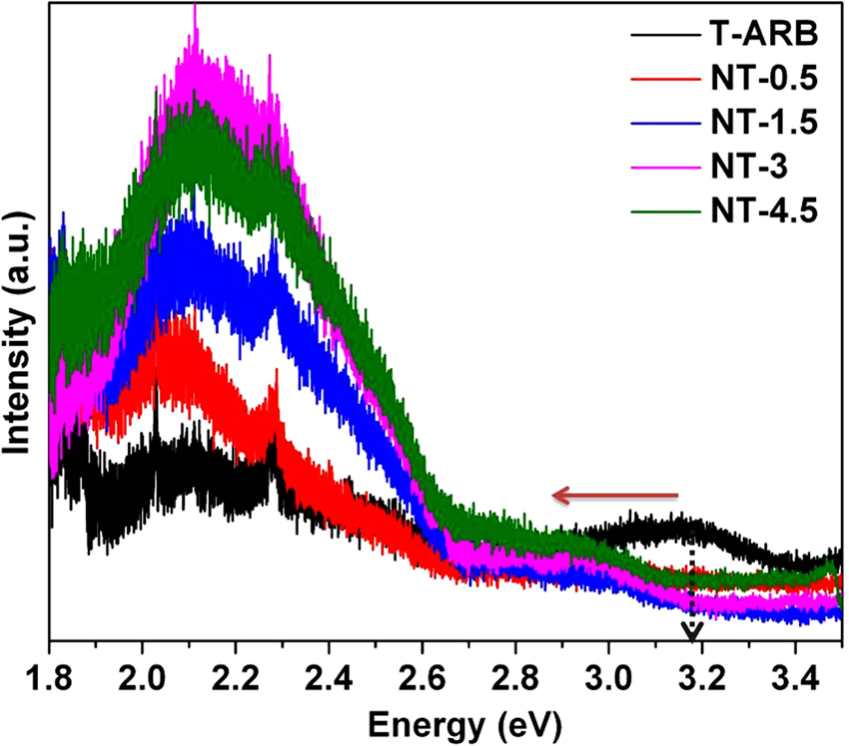
PL emission intensity of pristine and N-TiO_2_ polymorphs.

Photoluminescence spectra of pristine and nitrogen doped triphase TiO_2_ nanotubes are shown in Fig. 10. The PL spectra can be divided into two regions viz. region 1.8 to 2.7 eV and 2.7 to 3.4 eV. The PL intensity in the former region is due to the defects present in the samples, whereas the later is due to the band edge emission^41^. All samples exhibited a secondary peak emission in the range of 1.8 to 2.7 eV. The peak around 2.1 eV is due to oxygen vacancy related defects in addition to the under-coordinated Ti acting as electron traps^69,70^. The peak around 2.3 eV is due to the surface defects caused by oxygen deficiency and the associated Ti^3+^ ions^70–73^. For pristine TiO_2_, the intensities of secondary peaks are nearly equal to that of main emission peaks (around 3 eV). For nitrogen doped TiO_2_ polymorph the relative intensity of the secondary peaks is enhanced remarkably compared to the main emission peaks. This demonstrates the formation of oxygen defects in the TiO_2_ lattice as an effect of nitrogen doping. It is also observed that the defects in TiO_2_ increase as we increase the nitrogen doping concentration from 0.5 to 3 wt% and then it remains almost constant from 3 to 4.5 wt%. This study proves that the nitrogen doping is accompanied by the formation of oxygen vacancies. The higher intensity of N-TiO_2_ samples in this region compared to pristine TiO_2_ even though indicate high radiative recombination of charge pairs, the majority of non-radiative recombination that releases phonons is a major pathway for the photogenerated charge annihilation in TiO_2_ (as in indirect band gap semiconductors)^74^. It is already proved that N-TiO_2_ possesses better photocatalytic activity compared to pristine TiO_2_. Hence the results demonstrates that the N-TiO_2_ have a lower non-radiative recombination rate compared to pristine T-ARB.

When we compare the band edge emission region in the spectra (2.7 to 3.4 eV) for all the samples, it is observed that T-ARB has highest emission which is indicative of high recombination. It is observed that the main emission peak is shifted to lower energy for the nitrogen doped samples which indicates that there is a decrement in band gap (pointed in Fig. 10). The main emission peak of doped TiO_2_ polymorphs are in the order of NT-4.5 > NT-3 > NT-0.5 > NT-1.5. The low PL main emission intensity of nitrogen doped TiO_2_ is indicative of the forbidden recombination of photogenerated electrons and holes. It is clearly observed that NT-1.5 is having the lowest emission and the band edge emission peak intensity of all the samples inversely follows the trend observed in hydrogen generation efficiency ie lowest emission tends to higher hydrogen generation. This may be due to the formation of oxygen vacancies which actually served as electron capture traps, and hence separated the charge carriers and reduced the recombination significantly. However, higher nitrogen doping as in the case of NT-3 and NT-4.5 creates lot of vacancies which tends to behave as recombination centers hence increases the recombination rate thus lead to poor water splitting efficiency. The highest recombination is observed in NT-4.5 which may be due to either high nitrogen doping whose defect states act as recombination centers or due to the difference in number of phases present. It is already noted that the emission peak intensity due to defects in the former region of PL spectra of NT-4.5 and NT-3 are almost similar. Therefore the most probable reason for high band edge emission intensity of NT-4.5 is due to its biphase nature that decreases the rate of electron-hole pair separation hence increases the recombination when compared to NT-3. From the above results it is clear the defects created are saturated with increase in nitrogen doping (as in NT-3 and NT-4.5) but affect the phase composition leading to ARB to AR transformation.

## Discussion

**Figure 11:**
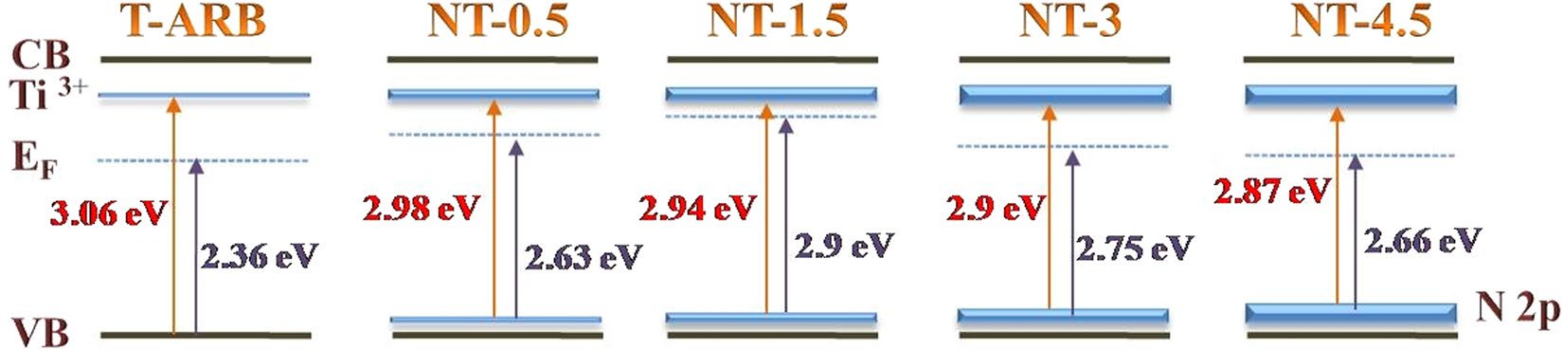
Effect of nitrogen doping in triphase TiO_2_ nanotubes.

Combining the results of the above analysis, a schematic diagram to illustrate the effect of nitrogen doping in triphasic TiO_2_ in comparison with undoped T-ARB has been derived and is depicted in Fig. 11. The abbreviations such as CB, VB and E_F_ correspond to the conduction band edge, valence band edge and Fermi level respectively. The growth of Ti^3+^ and N 2p states are clearly depicted in the figure. The presence of oxygen vacancies generated under the synthesis conditions in T-ARB gives rise to Ti^3+^ level below the CB. When T-ARB is doped with nitrogen as in NT-0.5 and NT-1.5, the Ti^3+^ state and N 2p state grow denser, leading to the decrement in band gap and increment in shift of E_F_ towards CB. At higher nitrogen doping, as in case of NT-3 and NT-4.5, the density of Ti^3+^ states reach a saturation level, as evident from O1s (Fig. 9a) and PL spectra (Fig. 10). The increase in density of the N 2p states leads to further decrement of band gap in these samples. However the Fermi level shifts away from the CB edge as seen from Mott Schottky analysis. This is due to the decrement in brookite phase concentration in NT-3 and its absence in NT-4.5.

**Figure 12:**
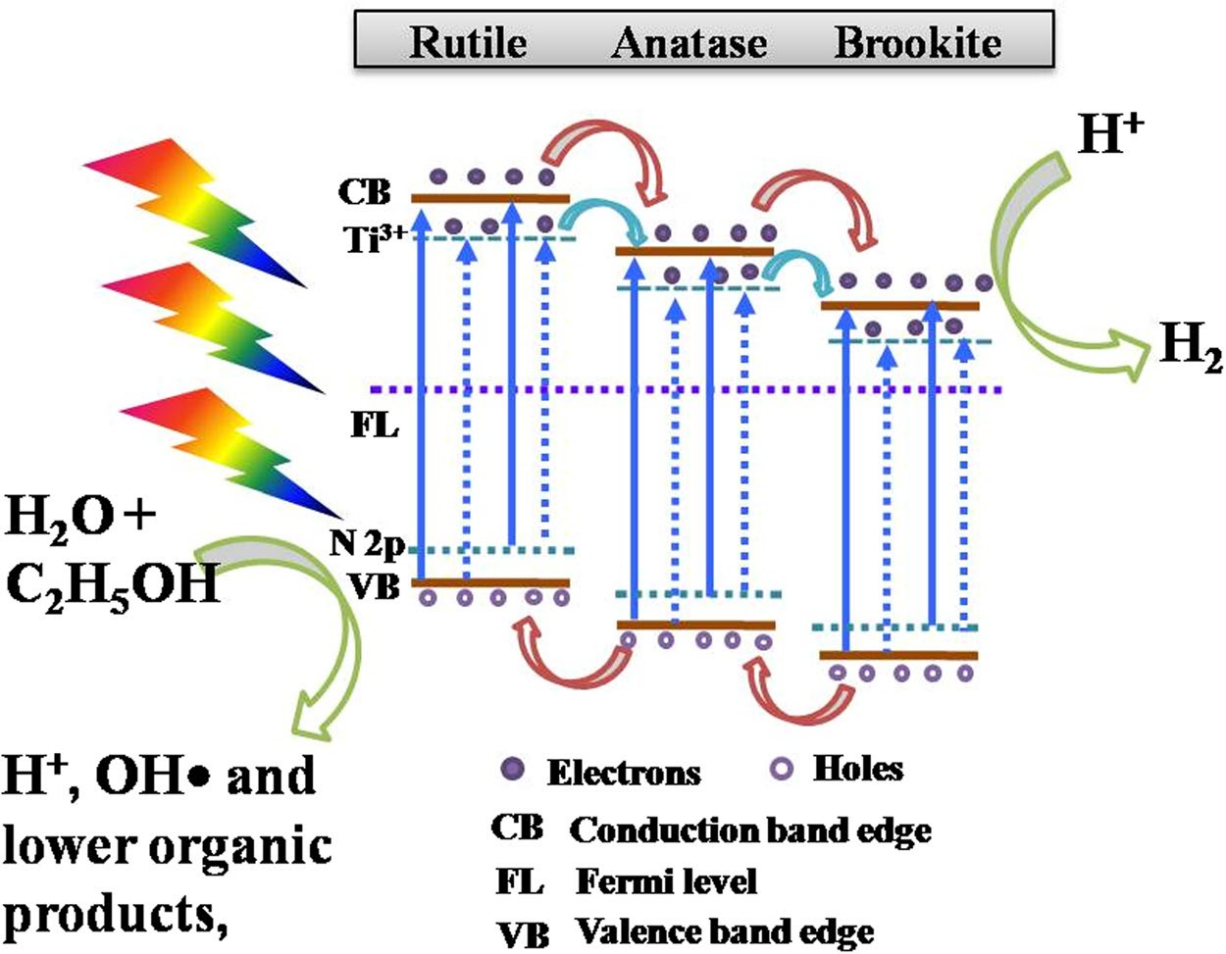
Schematic diagram for efficient charge transfer mechanism in NT-1.5.

From UPS analysis, it is clear that there is alteration in VB edge due to N doping. It is also clear that the nitrogen doping increased Ti^3+^ defects as confirmed from XPS and PL spectra analysis. Based on the above analysis of N-TiO_2_ in comparison with pristine T-ARB, the schematic for the effective photocatalytic charge transfer mechanism in NT-1.5 is proposed (Fig. 12). Because of the synthesis conditions and nitrogen doping, Ti^3+^ levels are formed below the CB edge and N 2p states appear above the VB edge of each phase as depicted in Fig. 12. When NT-1.5 is photo-irradiated, the electrons in VB and N 2p states of each phase absorb photoenergy and excited to their CB and Ti^3+^ states depending upon the absorbed photoenergy that varies from UV to Visible light.

Since the CB and VB edge positions of anatase, rutile and brookite are different; the Ti^3+^ and N 2p levels in one phase will be close to CB and VB of the adjacent phase respectively. This paves way for easy transfer of excited charge carriers thereby increasing the charge separation. The excited electron transfer pathway in triphase system is assumed to be from rutile to anatase to brookite^41^. The excited electrons in Ti^3+^ state of rutile phase transfers to anatase CB, whereas electrons in Ti^3+^ state of anatase transfers to brookite CB leading to the high life time of charge carriers which will be available for water splitting reactions. In addition the excited electron in the conduction band of each phase transfers to CB of adjacent phase. Similarly, the holes in N 2p and VB of one phase flow in reverse direction through VB and N 2p states of adjacent phase. Thus more number of charge carriers will be available for water splitting reactions. The presence of Ti^3+^ defect states and N 2p in triphase TiO_2_ appears to make a significant difference in catalytic acitivity. This is why we notice 12 times of hydrogen generation in NT-1.5 compared to pristine triphase TiO_2_ (T-ARB).

**Figure 13:**
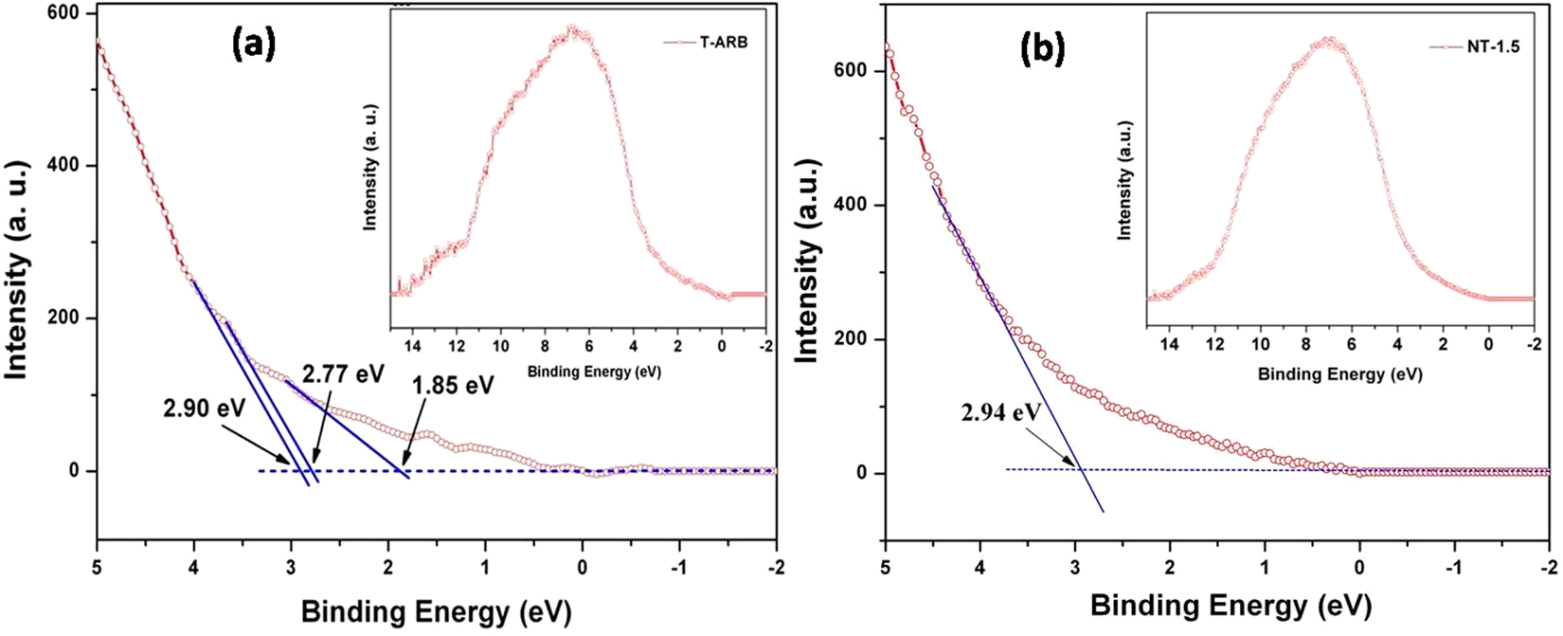
Synchrotron UPS spectra of (**a**) T-ARB; and (**b**) NT-1.5.

In our previous study on T-ARB^41^, the angle resolved photoelectron spectroscopy (ARPES) using synchrotron radiation with incident photon energy of 27 eV is used to probe its valence band features, whose results are represented here in Fig. 13a. Three leading edges (valence band onsets) of the valence band are observed. The points of intersections of the linearly extrapolated tangents along these edges with the binding energy axis are found to be 1.85, 2.71 and 2.9 eV. Each of these values corresponds to valence band edge of one of the three phases present in the sample. Thus synchrotron UPS fetched us a clear resolution in the VB edge features of triphasic T-ARB. Hence in order to distinguish the modified VB edge features, the synchrotron UPS is performed for NT-1.5 whose results are given in Fig. 13b. There is no clear resolution of three leading edge valence band onsets as observed in case of T-ARB (Fig. 12a). Only one VB onset value is obtained which is around 2.94 eV. The absence of clear resolution in the VB onsets in the UPS spectra corresponding to NT-1.5 in contrast to that of T-ARB is due to the presence of N 2p states above the valence band of each phase which are closely coupled as depicted in Fig. 12.

## Conclusion

In summary, nitrogen is doped in various concentrations in triphasic TiO_2_ nanotubes that induced phase transformation as well as oxygen vacancy defects. The detailed analysis of all the N-TiO_2_ nanotubes demonstrates that, excess nitrogen doping induces brookite to anatase phase transformation with increase in N 2p density of states. The defect formation is found to be saturated at excess N doping. Though photocatalytic reactivity difference is noticed among different N-TiO_2_ nanotubes, their overall efficiency is high compared to pristine triphasic TiO_2_ nanotubes. The hydrogen generation results indicate that nitrogen doped triphasic TiO_2_ nanotubes perform better than nitrogen doped biphasic nanotubes. The highest hydrogen generation obtained by NT-1.5 is due to the optimum nitrogen doping in triphase structure. The presence of Ti^3+^ states below the conduction band edges of each phase and N 2p states above the valence band edge of each phase reduced the band gap making it visible light active photocatalyst. In addition, it facilitated easy transfer of photo-excited charge carriers to the neighbor phase conduction band, thereby reducing recombination rate and enhancing water splitting efficiency. In general the present findings imply that optimal nitrogen doped multiphase TiO_2_ nanotubes can result in high quantum efficient visible light photocatalyst.

## Methods

### Synthesis of TiO_2_ nanotube powders

TiO_2_ nanotube powders are synthesized by potentiostatic rapid breakdown anodization technique using titanium and platinum foils as the working and counter electrodes, respectively, in 0.1 M perchloric acid electrolyte^2,21^. Before performing the experiment, the titanium foil (0.5 mm thickness, 99% pure, cut to the size of area 2.5 × 0.5 cm^2^) is polished smoothly to remove the oxide layer on the surface. The polished foils are ultrasonicated in alcohol, acetone followed by water and dried in N_2_ gas stream. A constant potential is applied across the electrodes (Pt mesh and Ti foil), kept 15 mm apart in 0.1 M HClO_4_, using a programmable DC power supply (Agilent N6700series). The synthesis is performed at 11 V, where 9.5 V is the threshold voltage for the rapid breakdown anodization (below which no TiO_2_ nanopowders are seen to fall into the solution)^41^.

The anodization process is continued till the whole titanium foil is etched as TiO_2_ powders. The TiO_2_ powders obtained are washed several times with de-ionized water, centrifuged and dried at 70 °C for 15 h. To synthesize nitrogen doped TiO_2_ nanotube powders, the nitrogen source (hydrazine hydrate) in various concentration (0.5, 1.5, 3 and 4.5 wt%) is added to the electrolyte (0.1 M HClO_4_) solution and the same synthesis procedure is followed. The pristine sample is labeled as T-ARB whereas the nitrogen doped TiO_2_ nanotubes (N-TiO_2_) synthesized at various hydrazine hydrate concentrations are labeled as NT-0.5, NT-1.5, NT-3 and NT-4.5 where the numbers represent the concentration of nitrogen source added.

### Materials Characterization

The morphological analysis of pristine and N-TiO_2_ nanopowder samples are performed by Field Emission Scanning Electron Microscope (FESEM), (JEOL 6360) and Transmission Electron Microscope (TEM) (JEOL 2010). Energy Dispersive Spectroscopy (EDS) microanalysis was carried out using an Apollo X Silicon Drift Detector attached to FEI make Helios NanoLab-600i dual beam field emission Scanning Electron Microscope (SEM). The samples for TEM studies are prepared by placing a drop of sample suspension in methanol on a carbon coated copper grid and allowing it to dry. The crystal structure of TiO_2_ nanotubes is analyzed by High Resolution TEM (HRTEM). The phase identifications of all the samples are carried out using X-Ray Diffractometer (D8, Bruker). An UV Vis spectrometer (PerkinElmer, Lambda 750 S) equipped with a 60 mm diameter integrated sphere in reflectance mode is used for band gap measurements. Photoluminescence studies are recorded using a photoluminescence spectrometer (Renishaw, UK), equipped with a confocal microscope having a “He-Cd laser” operating at 325 nm at a power level of 200 mW. A micro-Raman spectrometer (inVia, Renishaw, UK), in the back scattering configuration, with Ar + laser (514.5 nm) is used as excitation source, diffraction gratings of 1800 gr·mm^−1^ for monochromatization and a thermoelectric cooled charge coupled device (CCD) as detector to study the vibrational modes. The X-ray photoelectron spectra (XPS) were collected using hemispherical analyzer EA 15 (PREVAC) equipped with dual anode X-ray source RS 40B1 (PREVAC). The measurements were performed using Al Kα (1486.6 eV) radiation and analyzer pass energy of 100 eV. The electron binding energy (BE) scale was calibrated with adventitious C 1 s core level at 285.0 eV. The ultra-high vacuum (UHV) conditions of 1·10^−9^ mbar were maintained during the measurements. The ultraviolet photoelectron spectroscopy (UPS) data was recorded using the He discharge ultraviolet source UVS 40A2 (PREVAC), which produced the characteristic excitation line He Iα (21.2 eV) and analyser pass energy of 10 eV at a chamber pressure of lower than 10^−8^ mbar.

The valence band spectra were measured with angle resolved photoelectron spectroscopy (ARPES) beam line (BL-3) at Indus-1 synchrotron radiation source, RRCAT, Indore. Vacuum in the analysis chamber was better than 3 × 10^−9^ mbar during the measurements. The analysis chamber is equipped with SPECS PHOIBOS 150 electron analyzer. UPS spectrum from Au foil was used as a reference to characterize the monochromatic synchrotron light from the beamline.

### Hydrogen generation Studies

The water splitting experiments are carried out by dispersing 10 mg of the photocatalyst in ethanol-aqueous solution (1:4 ratio) in a quartz photocatalytic cell of 50 mL capacity and sealed with a rubber septum. The suspension is carefully purged with argon for 30 minutes. The quartz cell with dispersed sample is kept at 20 cm away from the solar simulator (equipped with 300 W Xenon arc lamp and AM 1.5 cut-off filter from Newport) to ensure one sun irradiation conditions^22^. The generated hydrogen is measured every hour by periodically withdrawing gas samples followed by quantification using a gas chromatograph (GC, Agilent 7690) for the total reaction time of 4 hours.

## Electronic supplementary material

Supporting information
