# Supplementary material for: A Study on Doped Heterojunctions in TiO2 Nanotubes: An Efficient Photocatalyst for Solar Water Splitting
Source: Sci Rep. 2017 Oct 30;7:14314. doi: 10.1038/s41598-017-14463-0 (PMC5662732; doi:10.1038/s41598-017-14463-0)
Supplement: Supplementary file 1 — Supporting information [file 41598_2017_14463_MOESM1_ESM.pdf]

Supporting Information

A Study on Doped Heterojunctions in TiO_2 Nanotubes: An Efficient Photocatalyst for Solar Water Splitting

Preethi L. K[1]*, Rajini P Antony[2], Tom Mathews[1]*, Lukasz Walczak[3], Chinnakonda S. Gopinath[4]

[1]*Surface and Nanoscience Division, Materials Science Group, Indira Gandhi Centre for Atomic Research, Homi Bhabha National Institute, Kalpakkam 603 102, India*

[2]*Chemistry Division, Chemistry Group, Bhabha Atomic Research Centre, Mumbai-400 085, India*

[3]*PREVAC sp. z o.o., Raciborska 61, 44-362 Rogow, Poland*

[4]*Catalysis Division, National Chemical Laboratory, Dr. Homi Bhabha Road, Pune 411 008, India*

**Email: preaag@gmail.com, tom@igcar.gov.in*

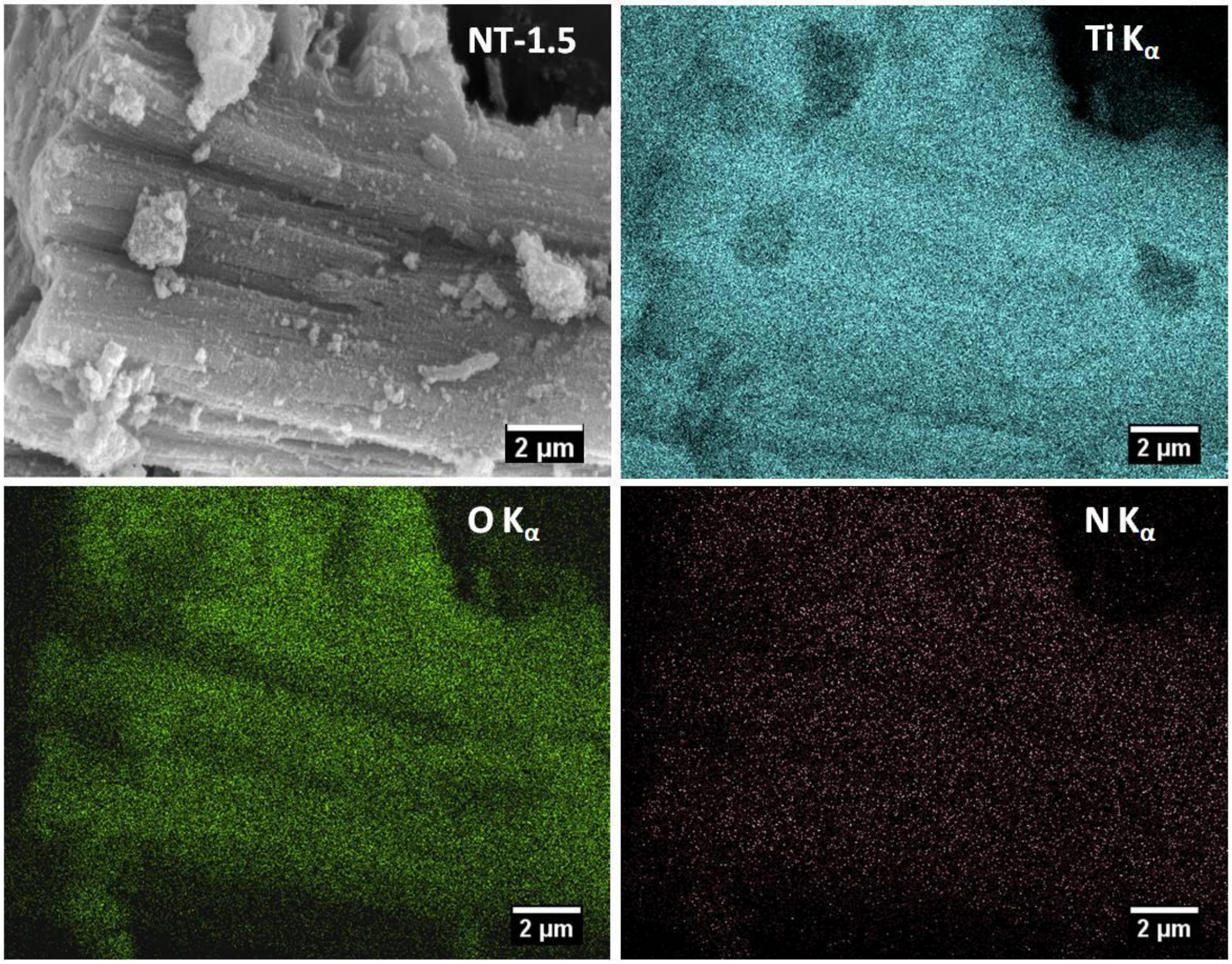

Figure S1. (a) Secondary electron (SE) micrograph of NT-1.5; (b, c, d) Ti K_α, O K_α and N K_α X-ray maps respectively corresponding to SE image given in (a).
